# A Dual-Branch Fusion Network Based on Reconstructed Transformer for Building Extraction in Remote Sensing Imagery

**DOI:** 10.3390/s24020365

**Published:** 2024-01-07

**Authors:** Yitong Wang, Shumin Wang, Aixia Dou

**Affiliations:** Institute of Earthquake Forecasting, China Earthquake Administration, Beijing 100036, China; wangyt@ief.ac.cn (Y.W.); douaixia@ief.ac.cn (A.D.)

**Keywords:** building extraction, convolutional neural network, deep learning, high resolution remote sensing imagery, dual-branch fusion, self-attention

## Abstract

Automatic extraction of building contours from high-resolution images is of great significance in the fields of urban planning, demographics, and disaster assessment. Network models based on convolutional neural network (CNN) and transformer technology have been widely used for semantic segmentation of buildings from high resolution remote sensing images (HRSI). However, the fixed geometric structure and the local receptive field of the convolutional kernel are not good at global feature extraction, and the transformer technique with self-attention mechanism introduces computational redundancies and extracts local feature details poorly in the process of modeling the global contextual information. In this paper, a dual-branch fused reconstructive transformer network, DFRTNet, is proposed for efficient and accurate building extraction. In the encoder, the traditional transformer is reconfigured by designing the local and global feature extraction module (LGFE); the branch of global feature extraction (GFE) performs dynamic range attention (DRA) based on the idea of top-k attention for extracting global features; furthermore, the branch of local feature extraction (LFE) is used to obtain fine-grained features. The multilayer perceptron (MLP) is employed to efficiently fuse the local and global features. In the decoder, a simple channel attention module (CAM) is used in the up-sampling part to enhance channel dimension features. Our network achieved the best segmentation accuracy on both the WHU and Massachusetts building datasets when compared to other mainstream and state-of-the-art methods.

## 1. Introduction

The extraction of building footprints is of great significance in the fields of urban development change monitoring, population spatial distribution statistics, and natural disaster risk assessment [1,2,3]. With the progress of high resolution remote sensing technology, it easier to obtain high resolution remote sensing image data with rich information [4]. With the rapid development of deep learning, especially the powerful feature extraction and expression abilities of deep learning models, there is great potential and application prospects in remote sensing building recognition [5,6].

Semantic image segmentation involves categorizing individual pixels within an image into predefined classes. The task becomes particularly challenging when dealing with high resolution images that contain intricate detail [7,8,9]. In an effort to enhance the precision of semantic segmentation for buildings, researchers have used robust linear fitting capabilities of convolutional neural networks (CNNs) for building extraction. Nevertheless, the fixed properties of convolution kernels and local receptive fields limit their effectiveness in capturing global features [10]. Based on this foundation, numerous CNN-based approaches aim to enhance their capacity to model global contextual information [11,12]; the common strategies involve constructing multi-scale features through feature pyramids, or constructing larger convolutional kernels to obtain larger receptive fields [13]. Recently, the advent of transformer technology [14] has catalyzed the rapid advancement of deep learning. Unlike convolution, transformer technology leverages the self-attention mechanism for feature extraction, excelling in modeling global contextual information. Its feature extraction and global contextual modeling capabilities surpass those of CNNs, making transformer-based networks instrumental in many tasks. Transformer-based networks have also achieved better results than CNNs in many computer vision tasks [15]. For example, Swin [16] obtained rich global contextual information through multi-scale self-attention. However, the transformer technique still has some problems in semantic segmentation tasks. On the one hand, due to the rich details of buildings obtained from high resolution remote sensing images whose complex contextual information can also be captured, the standard self-attention mechanism introduces redundant information [17]. On the other hand, it relies on a large amount of data for training in order for the model to reach convergence [18]. Due to the fact that the transformer maps image chunks into feature vectors, it is ineffective for extracting local detail information [19]. Therefore, it is necessary to design a reasonable network structure that can reduce the computational redundancy of the model while combining global and local information.

To mitigate computational redundancies within the transformer model and effectively integrate local and global features, a dual-branch fused reconstructive transformer network, DFRTNet, is proposed. The network adopts a four-scale encoding and decoding structure as a whole. Within the first three encoder scales, the traditional transformer network was refactored, and a local and global feature extraction (LGFE) module was designed for efficient extraction and fusion of local and global features. In the final encoder layer, a global self-attention module was employed to extract global features. Additionally, the decoder uses a simple channel attention module (CAM) for channel dimension enhancement after up-sampling splicing. More specifically, our LGFE module utilizes two branches, the global feature extraction (GFE) module and the local feature extraction (LFE) module. These components are refactored to reconfigure the traditional transformer encoder. Additionally, a basic multi-layer perceptron (MLP) module is utilized to facilitate feature fusion. GFE is based on the idea of top-k attention. It computes the relevance matrix via queries and keys on the global space, obtaining the first k most relevant regions in each row of the matrix, so that queries perform token-to-token self-attention computation only with key–value pairs of the first k most relevant regions, effectively mitigating computational redundancies. The LFE branch utilizes parallel multi-scale depth-separable convolution and max-pooling operations to acquire finely detailed local features. Furthermore, the local window self-attention was employed to reinforce these fine-grained local features at different scales. In conclusion, our proposed network approach was evaluated against mainstream and state-of-the-art methods using the WHU building dataset and the Massachusetts building dataset. The results demonstrate that our network exhibits an advantage over competing networks, while keeping the number of network parameters constant.

Overall, this article makes three main contributions:

1. A dual-branch fused reconstructive transformer network is proposed, which utilizes a dual-branch for fusing CNN and transformer approaches. This novel network adapts the conventional transformer model by integrating the local and global feature extraction (LGFE) module within the intermediate and shallower layers, facilitating the efficient extraction and effective fusion of both global context and local features.
2. The GFE module is designed to perform token-to-token computation based on the idea of top-k attention on the most relevant regions of the markers. This approach minimizes interference from irrelevant areas, while efficiently extracting global contextual features and reducing computational overhead. Concurrently, the LFE module was designed to compensate for the potential loss of local semantic information in the GFE module. The LFE module employs parallel convolution to generate multiple finely detailed local features, and utilizes local self-attention to facilitate semantic interactions among pixels at the same location.
3. Comparative evaluations of our network were conducted against mainstream and state-of-the-art methods using two public datasets: the WHU building dataset and the Massachusetts building dataset. The results demonstrate that our network outperforms other networks, while maintaining a consistent number of network parameters.

In this paper, the related research is discussed in Section 2. Our methodology is presented in Section 3. Experimental details and analyses are provided in Section 4, which includes information about the dataset and the results of our experiments comparing our network with others. In Section 5, we discuss the impact of different modules in the network on performance. Finally, we summarize the key findings of our study as well as our vision for future research in Section 6.

## 2. Related Research

The semantic segmentation of buildings holds significant importance in remote sensing. While convolutional neural networks (CNNs) are the primary technique used for this task, transformer-based approaches have gained prominence as a research focus [20], leading to the development of numerous transformer variants [21,22,23], as well as the combined structure of transformer and CNN [24,25,26]. In this section, the recent advancements in building semantic segmentation are delved into, encompassing CNN-based approaches, transformer-based approaches, and the fusion of CNN and transformer techniques.

### 2.1. CNN-Based Semantic Segmentation of Buildings

CNNs have emerged as the dominant technique for semantic segmentation in re-mote sensing. FCN [27] is notable for pioneering the use of full convolution for pixel-level prediction, which significantly advances the development of semantic segmentation models. However, the inherent constraint of FCN on the input image size results in stacked convolutional layers with limited receptive fields, thereby hindering the effective capture of global contextual information. To effectively capture and recover features, researchers have focused on both coding and decoding structure design and improving the ability to capture contextual global information.

In the design of encoding and decoding architecture, Unet [28] performs multi-scale feature extraction through a pyramidal encoder and a symmetric decoder, and utilizes the encoder-to-decoder hopping connection to semantically interact with the shallow and deep networks. SegNet [29] employs the VGG16 [30] as the encoder network, and uses pooling indexes computed in the encoder for up-sampling decoding as a way to capture multi-scale feature representations. This suggests that the integration of multi-scale features plays a crucial role in building semantic segmentation. In terms of obtaining global contextual semantic information, HRNet [31] establishes a parallel structured backbone network that maintains high resolution features throughout the process, and achieves strong semantic information and precise location information through information interaction between different branches. Deeplab [32,33] series utilizes atlas spatial pyramid pooling (ASPP) to collect contextual cues. ASPP includes parallel atlas convolution with different expansion rates [34] to obtain larger contextual information without increasing the number of parameters, and Deeplabv3+ [35] utilizes ASPP for contextual information acquisition at multiple scales through coding and decoding structures. The above methods acquire global contextual information at multiple scales by combining different coding and decoding structures, as well as parallel convolution. However, due to the inherent limitations of the convolutional kernel [10,36], the model receives limitations in its ability to capture contextual dependencies, resulting in suboptimal semantic segmentation results.

### 2.2. Transformer-Based Semantic Segmentation of Buildings

In the past few years, there has been significant progress in transformer technology for semantic segmentation [15]. VIT [14] first applies transformer to a computer vision task, which achieves good results in the image classification task by constructing a pure transformer with a sequence of image chunks as input. Meanwhile, the transformer-based semantic segmentation method has achieved good results in the GF3 images building extraction task [37]. However, the transformer-based architecture has two main problems: high computational load [38] and poor local information extraction [39]. Swin transformer [16] introduces the feature pyramid structure for the low output resolution of transformer models, and achieves good performance in semantic segmentation tasks while reducing the computational load. Previous studies [40] have confirmed that the swin transformer-based backbone network achieves better segmentation performance than MAP-Net [12]. Some experiments have also attempted to artificially design other attention windows, including spatially reduced attention [41], localized window attention [42], etc., to reduce the high computational and storage costs of transformer, and to improve its local feature extraction capabilities. Sparse token transformer (STT) [18] represents buildings as “sparse” feature vectors, enabling accurate building extraction with reduced computational effort by learning long-term token dependencies. PoolFormer [26] refactors the transformer structure by replacing the attention module with a simple spatial pooling operator (i.e., pooling), and performs the global feature extraction via a channel MLP. Currently, there are fewer pure transformer-based building extraction methods.

### 2.3. Semantic Segmentation of Buildings Based on CNN and Transformer

The inductive bias in the CNN architecture makes it lack a global long-range dependency representation in the image, and the transformer-based structure is ineffective for local feature extraction; thus, combining the CNN and transformer architecture may be an effective way to improve the performance of semantic segmentation [41,43]. TransUnet [25] connects transformer and Unet in tandem to become a powerful encoder for extracting image features, and transformer converts the labeled image blocks in the CNN feature map into an input sequence for global context extraction. Similarly, Swin transformer [16] can be used as an encoder in tandem with a CNN network decoder for building semantic segmentation tasks; finer segmentation results are obtained by applying boundary constraints to the segmentation results using auxiliary boundary detection branches [44]. sTransFuse [41] and TransFuse [45] adaptively fuse the decoder network through a feature aggregation module CNN branch and the feature mapping of the transformer branch. To address the issue of high computation for the transformers, the LiteST-Net [43] simplifies the matrices of keys, queries, and values into only one matrix of values. The LiteST-Net is summed with the features extracted from the CNN, and fully obtains the local features of buildings with global features. However, efficiently integrating the localization of convolution and the global correlation of transformer in the encoder is still the focus of current research.

## 3. Materials and Methods

**Figure 1:**
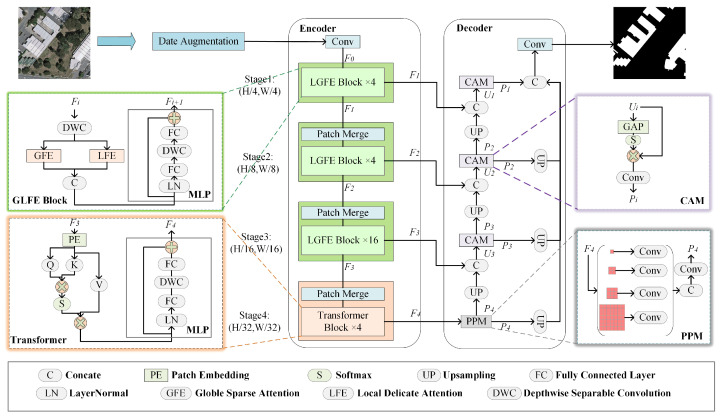
Overall architecture of the DFRTNet.

To fully utilize the advantages of CNN and transformer while reducing the computational effort of the model, an efficient transformer network based on the dual-branch fusion of CNN and transformer is proposed, DFRTNet. Figure 1 demonstrates the overall architecture of the proposed network, which employs the encoder–decoder structure. The encoder network mainly consists of a convolutional module, a three-level LGFE module that includes a global feature extraction (GFE) branch, a local feature extraction (LFE) branch, and an MLP branch, along with a transformer block. The decoder consists of a pyramid pooling module (PPM) and three channel augmentation modules (CAM). Specifically, the encoder first performs feature extraction on the remote sensing images, and reduces their resolution to alleviate the computational load on subsequent network inputs. Subsequently, a bottom-up pyramid structure is adopted so that the local features are extracted through four different scale stages, (H/4,W/4), (H/8,W/8), (H/16,W/16), and (H/32,W/32). In the first three stages, the GFE branches within the LGFE block perform feature embedding of the image blocks. The DRA module was designed to calculate the attentional relevance matrix by globally averaging the queries and keys within the coarse-grained regions, and preserves the indices of the top k most relevant locations in each row. This procedure yields the most pertinent key–value pairs within each region. Consequently, these key–value pairs, along with queries, undergo token-to-token self-attention computation to acquire global contextual information. The LFE branch firstly obtains local features with halved resolution at different scales through local maximum pooling and three depth-separated convolutions with three different convolution kernel sizes. Secondly, LFE rearranges the image elements with the same position according to the pixel positions, arranges the image elements with the same position sequentially in a 2 × 2 window, and obtains refined local features at the original resolution. Finally, the LFE utilizes the local self-attention to interact with the local semantic information of the image elements obtained at different scales within the local window. Subsequently, the features of the two branches are dimensionally spliced and transported to the MLP module so that the output features have global contextual information as well as local reinforcement information. The output of each stage is used as the input of the next stage through patch merging. In the last stage, the traditional transformer module is used to interact with the global contextual information. In the decoder part, after the high-level features output from the transformer are input to the PPM [46,47] module to obtain the multi-scale context information, they are up-sampled to be fused with the features of the previous stage and augmented in the channel dimension. Enhanced features are up-sampled to the first level of feature map size, and finally the segmentation map is obtained after performing sigmoid function computation through the output of the convolution module.

In the following, Section 3.1 and Section 3.2 detail the structure and workflow of the GFE and LFE branches in the first three stages of the encoder, and Section 3.3 details the workflow of the decoder.

### 3.1. The Structure of GFE

**Figure 2:**
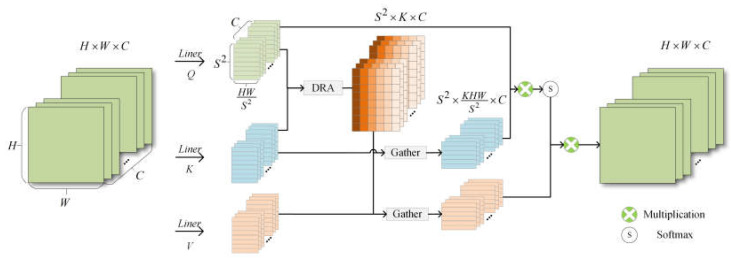
GFE branching structure.

In the self-attention computation of traditional transformer networks, sequencing image blocks generates keys, queries, and values via linear transformation. Queries determine the correlation between image blocks by computing the key–value pairs for the entire graph. The computational complexity quadratically scales with the number of image blocks. However, there is variability in the correlation of queries in different semantic regions for the key–value pairs across the entire graph. As a result, having each query compute tokens for the entire graph introduces computational redundancies. To address this issue, a DRA was designed. Unlike the traditional self-attention, the DRA module firstly computes the relevance weight matrix of queries and keys on a global scale. Secondly, the top k most relevant key–value pairs corresponding to each query from matrix are filtered. Finally, the query in each region calculates against its corresponding top k most relevant key–value pairs to reduce computational redundancies. The DRA was incorporated into the GFE module to establish global context dependency, as illustrated in Figure 2.

**Figure 3:**
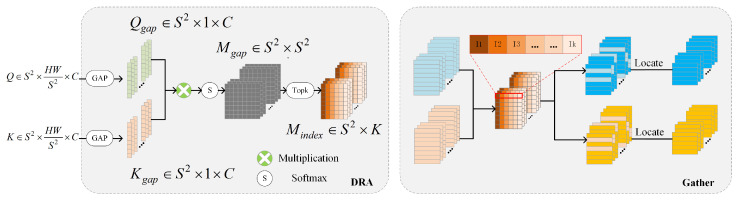
DRA structure and Gather process structure.

Taking the nth stage as an example, the feature $F_{n}\in R^{H\times W\times C}$ is divided without overlapping the sequence of image blocks $P_{n}\in R^{S^{2}\times \frac{HW}{S^{2}}\times C}$; the size of each image block is $\frac{HW}{S^{2}}$, and the number of image blocks $S^{2}$ is 8×8. Then, the region level query, key, and value, $Q,\;K,\;{V\in R}^{S^{2}\times \frac{HW}{S^{2}}\times C}$, respectively, are obtained via linear projection as follows:

(1) $$Q={P_{n}W}_{q}$$

(2) $$K={P_{n}W}_{k}$$

(3) $$V={P_{n}W}_{v}$$

where $W_{q}$, $W_{k}$, and $W_{v}$ in Equations (1)–(3), respectively, denote the three linear matrices of size C×C. Then, Q and K are input to the DRA module, as shown in Figure 3.

In the DRA module, Q and K perform a global average pooling (GAP) operation in the C dimension to obtain ${Q_{gap}\;and\;K_{gap}\in R}^{S^{2}\times C}$, respectively. $Q_{gap}$ and $K_{gap}$ characterize the overall features and the spatial distribution of Q and K within the region [48]. Then, $Q_{gap}$ and transposed $K_{gap}$ are multiplied to obtain the adjacency matrix ${M_{gap}\in R}^{S^{2}\times S^{2}}$, which is used to represent the region-to-region relevance [15]. $Q_{gap}$, $K_{gap}$, and $M_{gap}$ can be calculated as shown in Equations (4)–(6), respectively:

(4) $$Q_{gap}=GAP\left(Q\right)$$

(5) $$K_{gap}=GAP\left(K\right)$$

(6) $$M_{gap}=Q_{gap}{\left(K_{gap}\right)}^{T}$$

Subsequently, a neighbor matrix $M_{index}\in R^{S^{2}\times K}$ is used to represent the top k relevant region locations in each region. The *i*th row of $M_{index}$ denotes the index of the k most relevant positions corresponding to the *i*th query of Q. K and V gather the first k key–value pairs according to the region location index of $M_{index}$, and obtain ${K_{g}\;and\;V}_{g}\in R^{S^{2}\times \frac{KHW}{S^{2}}\times C}$, respectively. The Gather process is shown in the right panel of Figure 3, and $M_{index}$, $K_{g}$, and $V_{g}$ can be calculated, as shown in Equations (7)–(9), respectively:

(7) $$M_{index}=Topk\left(M_{gap}\right)$$

(8) $$K_{g}=Gather\left(Q,M_{index}\right)$$

(9) $$V_{g}=Gather\left(V,M_{index}\right)$$

where Topk(·) denotes the index at which the first k largest values are recorded on each row of the matrix $M_{gap}$, and Gather(∙) denotes the extraction of values from Q, K according to the index position matrix to obtain the most relevant key–value pairs $K_{g}$, $V_{g}$.

Finally, Q, $K_{g}$, and $V_{g}$ perform token-to-token multi-head self-attention computation to obtain features $O_{n}\in R^{S^{2}\times \frac{HW}{S^{2}}\times C}$ with global contextual information interaction, as shown in Equations (10) and (11):

(10) $$SA\left(Q,K_{g},V_{g}\right)=softmax\left(\frac{Q{\left(K_{g}\right)}^{T}}{\sqrt{d}}\right)V_{g}$$

(11) $$O_{n}=concate\left[SA\left(Q^{1},K_{g}^{1},V_{g}^{1}\right),\ldots ,SA\left(Q^{m},K_{g}^{m},V_{g}^{m}\right)\right]W^{o}$$

$O_{n}$ is reshaped to the input feature size $T_{n}\in R^{H\times W\times C}$. The computational complexity of traditional self-attention is $\Omega (2{\left(HW\right)}^{2}C)$. Among the computational complexity of GFE, the computational complexity of DRA is $\Omega ({{2(S}^{2})}^{2}C)$. The calculation complexity of multi-head attention is $\Omega (2HWk\frac{HW}{S^{2}}C)$. The total computational complexity is $\Omega ({{2(S}^{2})}^{2}C+2HWk\frac{HW}{S^{2}}C)$, which is lower than that of traditional self-attention. By computing the DRA module, the complexity of the traditional transformer self-attention computation can be reduced, while the global long-range dependency of the image is effectively established.

### 3.2. The Structure of LFE

**Figure 4:**
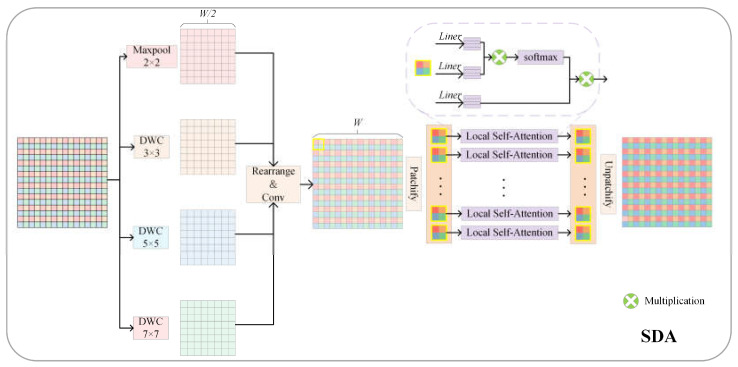
The structure of LFE.

To strengthen the network’s representation of the local feature information, the LFE branch was designed parallel to the GFE. Considering the effectiveness of depthwise separable convolution in extracting local features and the sensitivity of max pooling to the most significant feature information, LFE obtains local features of four different scale receptive fields through three depth-separable convolutions and max pooling. Then, LFE reorganizes the four local features to obtain the refined local features. Finally, LFE applies local window self-attention to the reorganized region to strengthen it. The LFE is shown in Figure 4.

Taking the nth stage as an example, four parallel efficient convolutional branches for the feature map $F_{n}\in R^{H\times W\times C}$ are used to extract the local feature information of different receptive fields, which are Maxpool(·) operation and three depth-separable convolutions DWC(·) with convolution kernel sizes of 3 × 3, 5 × 5, and 7 × 7 [49]. Through four convolutional branches, the local features $f_{n}^{1},\;f_{n}^{2},\;f_{n}^{3},\;and\;f_{n}^{4}\in R^{\frac{H\times W\times C}{4}}$ of the four branches are obtained, the resolution of the features is reduced to half. They can be calculated as shown in Equations (12)–(15), respectively:

(12) $$f_{n}^{1}={Maxpool}_{2\times 2}\left(F_{n}\right)$$

(13) $$f_{n}^{2}={DWC}_{3\times 3}\left(F_{n}\right)$$

(14) $$f_{n}^{3}={DWC}_{5\times 5}\left(F_{n}\right)$$

(15) $$f_{n}^{4}={DWC}_{7\times 7}\left(F_{n}\right)$$

Subsequently, pixel position rearrangement rearrange(·) is performed on the four local features [50] to obtain a feature map $f_{n}\in R^{H\times W\times C}$. To strengthen the local information interaction between the reorganized pixels, the local self-attention [51] LSA(·) with a window size of 2 × 2 is applied to $f_{n}$ to obtain fine-grained local features $L_{n}\in R^{H\times W\times C}$. Pixel rearrangement and local information interaction can be expressed by the following Equations (16) and (17), respectively:

(16) $$f_{n}={conv}_{3\times 3}\left(rearrange\left(f_{n}^{1},f_{n}^{2},f_{n}^{3},f_{n}^{4}\right)\right)$$

(17) $$L_{n}={LSA}_{2\times 2}\left(f_{n}\right)$$

Finally, the features $T_{n}$ and $L_{n}$ of the two branches are spliced in channel dimension. After layer normal (LN), they are input to the MLP module for global and local feature enhancement, and reduced to the original feature map channel size $F_{n}$. MLP includes an ${FC}_{1}$ layer to reduce the channel dimensions, a depth-separable convolution, and an ${FC}_{2}$ layer with the same channel. The computational process of the MLP module is shown in Equation (18):

(18) $$O_{n}=concate\left[SA\left(Q^{1},K_{g}^{1},V_{g}^{1}\right),\ldots ,SA\left(Q^{m},K_{g}^{m},V_{g}^{m}\right)\right]W^{o}$$

### 3.3. Decoder Network Structure

In the decoder network, the feature $F_{4}$ output from the last layer of the encoder passes through the PPM [44,47]. PPM utilizes the average pooled features of different subregions for multi-scale global contextual information. Subsequently, the module performs a depth-separable convolution (DWC) for dimensionality reduction to obtain $P_{4}$, which is shown in Equation (19):

(19) $$P_{4}=DWC\left(PPM\left(F_{4}\right)\right)$$

The features $F_{n}(n\in \left\{1,2,3\right\})$ output from the first three stages in the encoder are obtained, and $P_{n-1}$ is up-sampled and spliced with $F_{n}$. Considering the difference in semantic information between channels after feature fusion from the different stages, the spliced features are input to the CAM [52] module. The global max-pooling GMP(·) and a SoftMax operations are performed to obtain the weight $U_{n}$ of the features in the channel dimension. Then, $U_{n}$ is dot-multiplied with the spliced features to obtain the channel-enhanced features. The computation process of the first three stages is shown in Equations (20) and (21):

(20) $$U_{n}=softmax\left(GMP\left(concate\left[Up\left(P_{n-1}\right),F_{n}\right]\right)\right)$$

(21) $$P_{n}=conv\left(concate\left[Up\left(P_{n-1}\right),F_{n}\right]\times \left(1+U_{n}\right)\right)$$

Finally, the features $\left\{P_{1},P_{2},P_{3},P_{4}\right\}$ are up-sampled to the output feature size and dimensionally spliced. The final segmentation map is obtained after convolution and sigmoid(∙) computations.

### 3.4. Loss Function

In this paper, the cross-entropy loss and the dice loss functions [53] are selected to combine the function of $L_{total}$ to optimize the predicted value in the training process. The network model is solved in the training process of obtaining the loss value when the value of the weight parameter ω is known. The function is shown in Equation (22), and the weight of each loss function is set to 0.5:

(22) $$argmin(L_{total}|\omega )=argmin(0.5{\times L}_{ce}+0.5\times L_{D}|\omega )$$

where $L_{ce}$ is the cross-entropy loss function and $L_{D}$ is the dice loss function.

The cross-entropy loss function $L_{ce}$ is defined as shown in Equation (23):

(23) $$L_{ce}=\frac{1}{N}\sum_{i}L_{i}=\frac{1}{N}\sum_{i}-\frac{1}{N}\sum_{c=1}^{C}y_{i}lg\left(p_{i}\right)$$

where C denotes the number of categories, $y_{i}$ indicates whether it belongs to the positive class—if it belongs, $y_{i}$ is 1; otherwise, $y_{i}$ is 0. The $p_{i}$ denotes the probability value that the sample i belongs to category C. In this research, the category number C is 1. $L_{ce}$ is used to evaluate the loss incurred when categorizing pixel points during segmentation of an image. $L_{ce}$ can measure the degree of 0 difference between two different probability distributions of the same random variable; a smaller value of the function indicates that the two probability distributions are more similar, thus the better the prediction effect of the model.

The dice loss function $L_{D}$ is defined as shown in Equation (24):

(24) $$L_{D}=1-\frac{2\left|x⋂y\right|}{\left|x\right|+\left|y\right|}$$

where $\left|x⋂y\right|$ denotes the intersection of true and predicted samples, and $\left|x\right|+\left|y\right|$ denotes the concatenation of true and predicted samples, respectively; $\left|x\right|$ and $\left|y\right|$ denote the numbers of true and predicted elements of the sample, respectively. $L_{D}$ is a metric loss used to evaluate the similarity of the set between the predicted image and the real image.

## 4. Experimental Results and Analyses

### 4.1. Datasets

To evaluate the performance of the proposed network, extensive experiments on two representative datasets were conducted.

#### 4.1.1. WHU Building Dataset

The WHU dataset [54] is a large building dataset composed of remote sensing images from multiple sources, mainly including aerial and satellite images. Among them, there are 8819 aerial images (spatial resolution down-sampled to 0.3 m; each image is 512 × 512 pixels in size) covering a ground area of about 450 km^2^, and there are 17,388 satellite images with a spatial resolution of approximately 2.7 m, covering a ground area of about 550 km^2^. The whole sample labels of the building dataset are divided into two categories: building and background. In this research, 65% of the images in the dataset were randomly selected as the training set, 5% of the images as the validation set, and the remaining 30% of the images as the test set; these were used to train and test the network’s building extraction capabilities.

#### 4.1.2. Massachusetts Building Dataset

The Massachusetts buildings dataset [55] covers approximately 20,080 buildings of different scales and sizes in urban and suburban areas of the Boston region of the United States. The dataset consists of 151 high resolution remote sensing images, each with a size of 1500 × 1500 pixels and a resolution of 1.0 m, covering a ground area of about 340 km^2^ After overlap cropping (overlap of 128 pixels), an image dataset with an image size of 512 × 512 pixels was obtained. Among them, 3000, 200, and 1200 images were randomly selected to join the training, evaluation, and test sets, respectively.

### 4.2. Evaluation Indicators

In this study, four metrics were chosen, accuracy (Acc), recall (R), precision (P), F1 score (F1), and intersection over union (IoU), in order to evaluate the performance of our method and other SOTA methods. Their definitions are shown in Equations (25)–(29), as follows:

(25) $$Acc=\frac{TP+FN}{\left(TP+TN+FP+FN\right)}$$

(26) $$R=\frac{TP}{TP+FN}$$

(27) $$P=\frac{TP}{TP+FP}$$

(28) $$IoU=\frac{TP}{TP+FP+FN}$$

(29) $$F1=2\times \frac{Precision\times Recall}{Precision+Recall}$$

where TP denotes the number of labeled building image elements predicted as building image elements; FN denotes the number of labeled background image elements and predicted as background image elements; FP denotes the number of labeled background image elements predicted as building image elements; and TN denotes the number of labeled building image elements predicted as background image elements.

### 4.3. Experimental Setup

The proposed model was implemented based on the PyTorch framework, and all of the experiments were conducted on a single NVIDIA A30 Tensor Core GPU for 160 k iterations. We implemented an early stopping strategy, structured in 200 iteration cycles. During network training, if the loss in a subsequent cycle did not decrease compared to the preceding cycle, the training was halted. For the training of the two datasets, the WHU dataset and Massachusetts dataset, we used the Adamw optimizer [56] with a momentum of 0.9 and a weight value decay of 0.0001. The initial learning rate was set to 0.0001, which decayed in powers of 0.9 by the poly learning rate strategy. The batch size was set to 2, and the weight decay was set to 0.0001. In addition, certain data enhancement strategies are adopted for the training of the model: (1) random flipping; (2) random zoom-in and zoom-out cropping of the image at a ratio range of (0.5, 2); and (3) normalization of each channel of the image by calculating the mean and variance of the dataset.

### 4.4. Results of the Experiment

To evaluate the effectiveness of the network models proposed in this paper, the comparative experiments were conducted on several classical semantic segmentation methods using the WHU building dataset and the Massachusetts dataset, including the following CNN-based semantic segmentation models: Segnet [29], DeepLabv3+ [35], and HRNet [31], as well as the following transformer semantic segmentation models: TransUnet [25], Swin-T [16], and PoolFormer [26]. All of the network models were tested under the same experimental conditions.

#### 4.4.1. WHU Building Dataset

**Figure 5:**
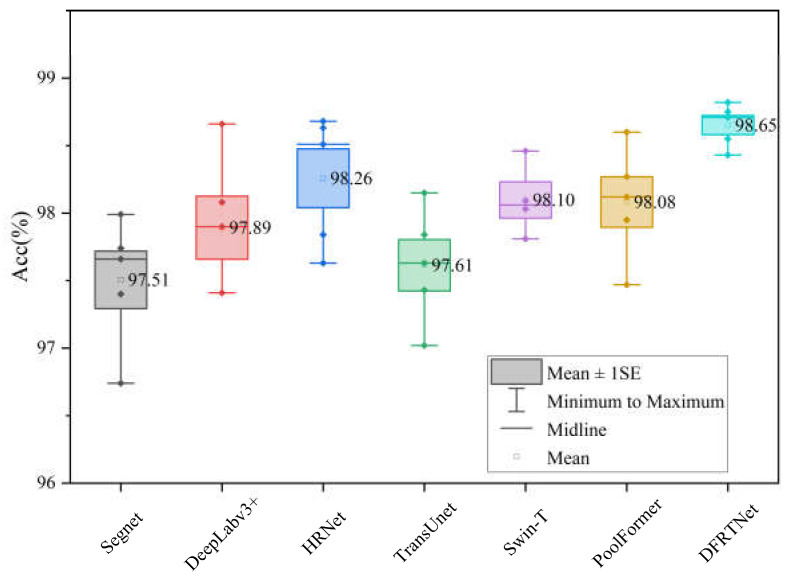
Accuracies of different networks under five-fold cross-validation experiment on WHU building dataset. The label ‘♦’ represents the accuracy value of each experiment.

To support the authenticity of model performance, we used a five-fold cross-validation method to verify each network. We divided the training set and validation set into five non-overlapping and equal-numbered parts. For each experiment, one piece of five datasets was selected as the validation set, and the remaining four pieces were used as the training set. Different networks used the same datasets in the same fold of experiments. We performed statistical analysis on the accuracy of five-fold cross-validation, as shown in Figure 5. The labels in the box plots represent the average accuracy values of five experiments.

As can be seen from Figure 5, the average accuracy achieved by DFRTNet in the five-fold cross-validation experiment is higher than those of the other networks. At the same time, the standard deviation (SE) of the accuracy obtained by DFRTNet is also smaller than those of the other networks, which proves that the dispersion degree of the accuracies obtained by DFRTNet at different folds is lower than those of the other networks.

Under the same experimental conditions, the evaluation indexes for the WHU dataset were obtained as shown in Table 1. It can be seen that all of the models in the table achieve good results, with our network having an advantage over the CNN-based models. Compared with SOTA method HRNet, our network obtained 4.93%, 5.07%, and 3.01% improvements in IoU, R, and F1, respectively, as well as 5.16%, 4.83%, and 3.16% improvements compared to DeepLabv3+, respectively. In the transformer-based model, relative to the standard Swin-T, our network improves the IoU, R, and F1 by 5.72%, 4.72%, and 3.52%, respectively. Compared to the TransUnet-S and PoolFormer-m48 with larger parameter counts, our network improves the IoU accuracies by 2.64% and 3.52%, respectively. From the quantitative results, our network has a clear advantage on the WHU dataset. Meanwhile, we found that the three metrics of the transformer-based networks were low compared to the CNN-based networks; the reason for this result may be that the small batch size makes it impossible for transformer’s network to converge with the same number of iterations, whereas our network has a faster convergence due to the addition of the convolutional module.

**Figure 6:**
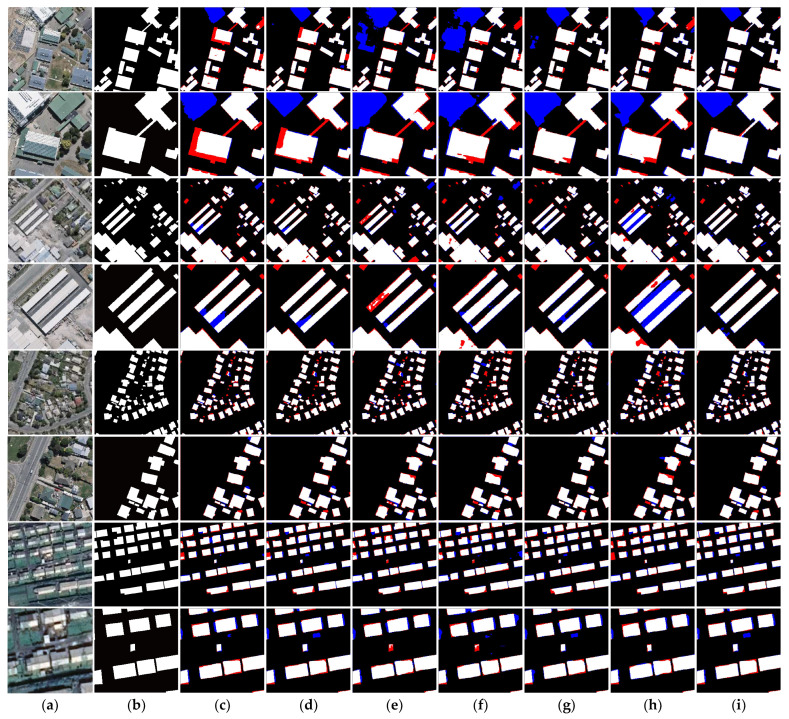
Visualization of the prediction results of different network models on the WHU building dataset. (**a**) Raw image; (**b**) ground truth; (**c**) Segnet; (**d**) DeepLabV3+; (**e**) HRNet; (**f**) Swin; (**g**) Transnet; (**h**) PoolFormer; (**i**) ours.

A visual comparison of the prediction results for different models on the WHU dataset from a qualitative point of view is shown in Figure 6. The first two columns of the subfigures represent the input images and real samples. The latter seven columns of the subfigures represent the prediction results of CNN-based Segnet and HRNet; transformer-based Swin, TransUnet, and PoolFormer; and our network, respectively. The even subfigure rows represent localized enlargements of the previous subfigure rows. We overlaid the prediction results of different networks with the real labels, and set the missed building pixels to red, the pixels incorrectly identified as buildings to blue, and the correctly identified buildings pixels to white. It can be seen that our method has better performance than the other methods. For example, compared with the other methods, our network is able to extract more complete and clearly bounded complex buildings, has more accurate localization for small buildings, and also achieves better recognition results than the other networks for buildings obscured by trees or shadows. These results indicate that the effective combination of the LFE module and the GFE module enables the network to extract richer details and global features.

#### 4.4.2. Massachusetts Building Dataset

**Figure 7:**
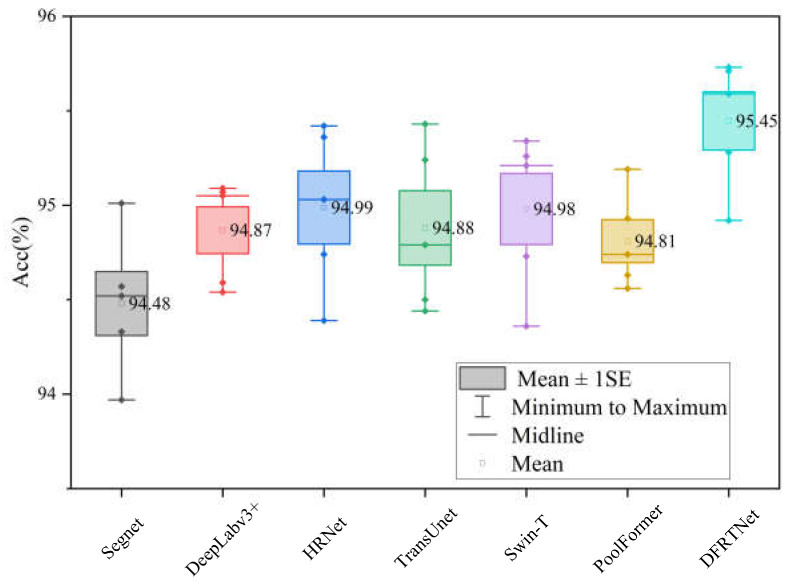
Accuracies of different networks under five-fold cross-validation experiment on Massachusetts building dataset. The label ‘♦’ represents the accuracy value of each experiment.

We performed the five-fold cross-validation experiment on the Massachusetts dataset and carried out statistical analysis on the accuracy metrics. As shown in Figure 7, the labels in the box plots represent the average accuracy values of five experiments. The average accuracy achieved by DFRTNet in the five-fold cross-validation experiment is higher than that of the other networks.

The evaluation results from each network in the Massachusetts buildings dataset are shown in Table 2, with bold text denoting the best-performing evaluation metrics on the corresponding dataset. Compared to the other methods, our proposed network also shows significant superiority on the Massachusetts buildings dataset. Relative to CNN-based DeepLabv3+, our network obtained 4.19%, 4.02%, and 2.46% improvements in IoU, R, and F1, respectively, and achieved 3.82%, 2.83%, and 2.17% improvements relative to transformer-based Swin-T.

**Figure 8:**
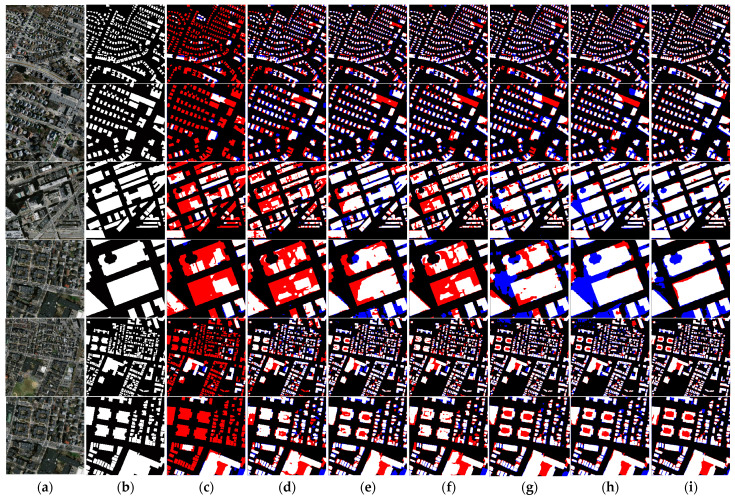
Visualization of the prediction results of different network models on the Massachusetts buildings dataset. (**a**) Raw image; (**b**) ground truth; (**c**) Segnet; (**d**) DeepLabV3+; (**e**) HRNet; (**f**) Swin; (**g**) TransUnet; (**h**) PoolFormer; (**i**) ours.

Through qualitative analysis, we can see from Figure 8 that for dense buildings, the prediction results of our network are more complete compared to the other networks, and the buildings that are covered by the shadows of high-rise buildings can also be recognized effectively. Meanwhile, we found that the difference between Swin-T based on transformer and Deeplabv3+ based on CNN is not large in terms of prediction results and accuracy metrics. We believe this was due to the fact that the transformer model did not reach effective convergence due to the number of iterations and the small batch size in this experiment. It further illustrates the robustness of our network’s framework design in extracting complex buildings.

### 4.5. Analysis of Complexity

To verify the relationship between the performance and complexity of the network, two metrics were used, the number of model parameters (Params) and the average time per iteration, for network complexity assessment with the other SOTA methods. The IoU metrics were used for the comparison of network performance. As shown in Table 3, although Segnet has the smallest number of parameters, the IoU is much lower than that of our model. In the transformer-based model, the complexity of our model in terms of the number of parameters is comparable to that of Swin-T, but the average time per iteration and the performance are significantly better than that of Swin-T. These results reaffirm that the performance advantage of our network is determined by the advantages of the network model architecture as a whole.

**Figure 9:**
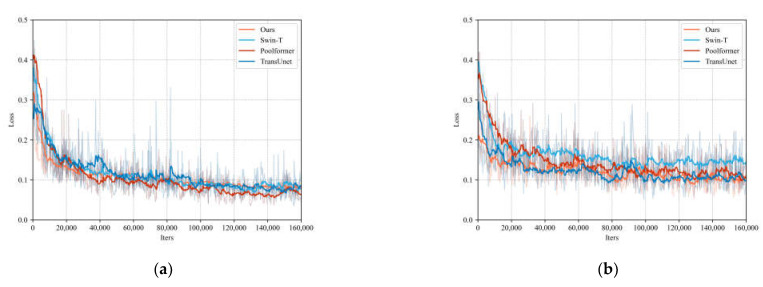
Visualization of loss curves for different network models. (**a**) Loss curves on WHU dataset. (**b**) loss curves on Massachusetts dataset.

Additionally, we visualized the training loss curves of our network alongside those of transformer-based networks, as illustrated in Figure 9. This visualization demonstrates that our network’s loss on both the WHU and Massachusetts datasets decreases more rapidly compared to the other network models. In the WHU dataset, DFRTNet reached convergence at iteration 120608, and the other networks also reached convergence. In the Massachusetts dataset, due to the small size of the dataset, TransUnet and Swin did not show early stopping at the specified iterations. DFRTNet reached convergence at iteration 150206, which also shows that the performance of DFRTNet is better than that of the transformer-based networks. Moreover, the magnitude of oscillations in our network’s loss is notably smaller than those observed in the other networks. This evidence further substantiates the effectiveness of our network model.

### 4.6. Analysis of Feature Visualization 

**Figure 10:**
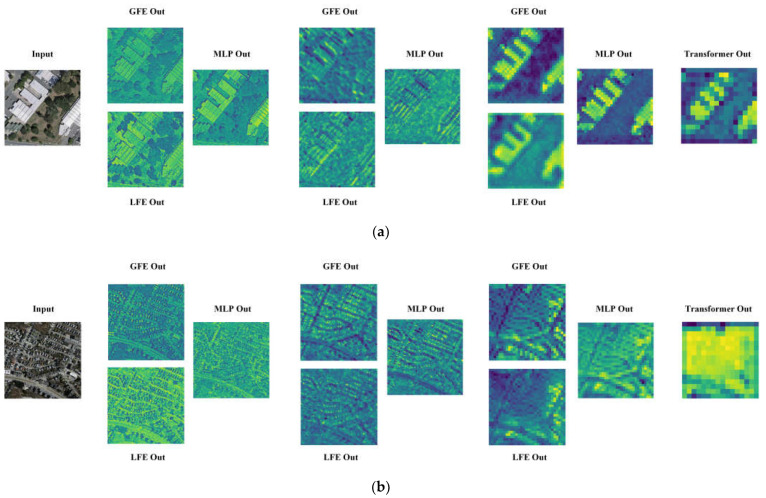
Visualization of output features for each module in the network encoder. (**a**) The output features of the image with large buildings. (**b**) The output features of the image with intensive buildings.

To obtain a more intuitive understanding of the representative feature information obtained by the two modules in the network, the features of the network were visualized. First, we visualized the output features of the LFE, GFE, and transformer modules of the model encoder at each stage. We zoomed in on the lower spatial resolution deeper features with linear interpolation. In the visualizations (Figure 10), the highlighted colors indicate the regions that the model pays more attention to, and the darker colors indicate the regions that the model pays less attention to. From the visualization in Figure 10, we can clearly see the output features of the input image after extraction by the two modules LFE and GFE, MLP-enhanced expression and output by the global self-attention module, respectively. In the two branches of the encoder, the model effectively extracts the local and global feature information of the input image, respectively. LFE pays more attention to the local detail information of the features, while GFE pays more attention to the overall distribution of the features. After MLP enhancement, the fused features provide enhanced expression of the global and local features. The network was able to pay attention to the information related to the two features, and suppressed the unimportant information. As the network deepens, the global information obtained by the LFE module and the GFE module in deeper layers (e.g., the third stage) is richer. At this time, features pass through the global self-attention module and interact with the global contextual information through the global self-attention. This proves the effectiveness of our proposed model.

**Figure 11:**
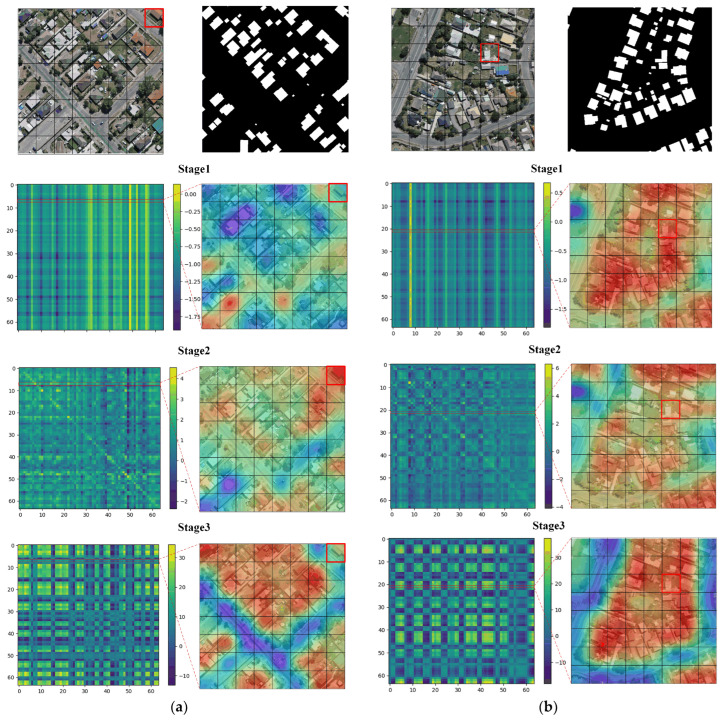

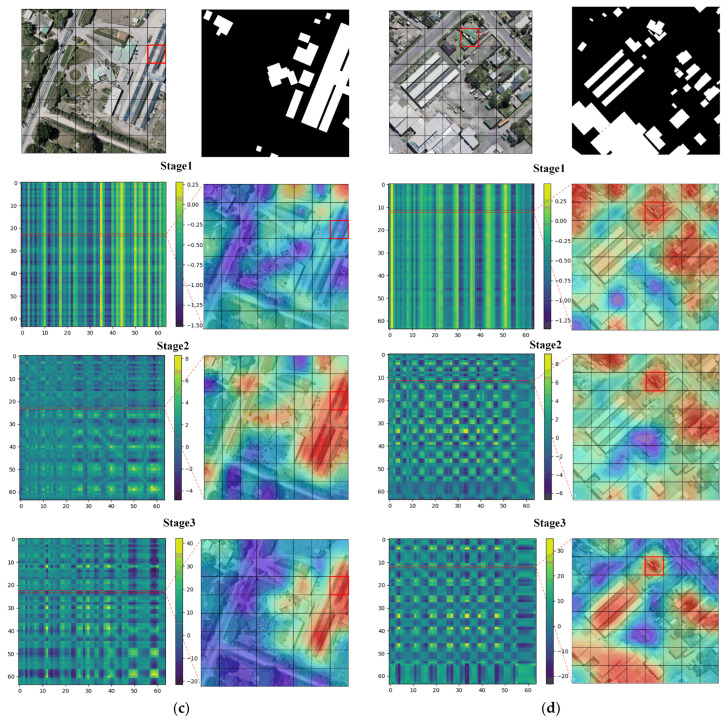
Attention map visualizations of GFE modules. (**a**–**d**) represent attention maps of four different building images. Red boxes represent the query area.

We also visualized the attention map of the GFE module. Unlike the direct output of the correlation matrix obtained from the computation of queries and keys, we took one image block containing the building in the attention map as the query; then, we visualized the relevance of the query to the whole image. We reshaped the *i*th row of the relevance matrix to the image block region size, which represents the relevance weight of the query of the *i*th image block to the keys of the whole image block. Subsequently, we used linear interpolation on the input image size, i.e., we obtained the attention heat map with the *i*th image block as the query. We selected four images for visualization, as shown in Figure 11a–d, where the odd columns represent the relevance weight maps and the even columns represent the heat maps corresponding to the query image blocks. We selected the image block containing the building as the query and indicated it with a red box. The red color in the heat map represents high relevance, while the blue color represents irrelevance. From Figure 11, we can see that under the DRA of the first stage, image block areas sparsely associated with buildings show highlighted values. With the deepening of the stage, the high correlation regions are more intense and accurate until they cover all of the relevant building regions in the whole map. This demonstrates that our GFE module is able to effectively extract global contextual information through DRA for shallow features.

## 5. Discussion

In order to explore the contributions of different modules as well as hyperparameter settings to our network encoder and decoder, we conducted two sets of ablation experiments on the WHU building dataset.

### 5.1. Impact of Different Modules

First, to explore the contributions and impact of different modules on the network encoder and decoder, we conducted ablation experiments on the WHU building dataset with different combinations of modules. Specifically, our network encoder mainly consists of the LFE and GFE in LGFE and the transformer module in the last stage, while the decoder mainly consists of the CAM module. The ablation experiments in the decoder part involve the CAM module. We performed four ablation experiments. LFE means that in the encoder part, the features only go through the LFE module and MLP. GFE means that in the encoder part, the features only go through the GFE module and MLP. GFE + LFE means that we did not use the CAM module on the basis of our network, and GFE + CAM and LFE + CAM mean that in the decoder part, the features go through the MLP output on the basis of the first and second sets of experiments, respectively. The accuracy metrics between the modules are shown in Table 4.

From the results of Table 4, it can be seen that the best scores of our model on each metric indicate the advantages of different modules in terms of performance. In particular, the segmentation performance is slightly improved when we add GFE and LFE (i.e., GFE + LFE). In addition, the last three experimental results in the table show that adding the CAM module is effective.

### 5.2. Impact of Hyperparameters

In the GFE module of the model’s encoder section, we performed ablation experiments to verify the necessity of the parameter *k* selection. The values of *k* we chose in the first three stages are {1, 4, 16}, with the purpose of keeping the number of image blocks involved in the computation in each stage relatively stable. Specifically, the computations of the image blocks in the first three stages were {$\frac{128\times 128}{8^{2}}\times K,\;\;\frac{64\times 64}{8^{2}}\times K,\;\;\frac{32\times 32}{8^{2}}\times K$}, and the corresponding numbers of image blocks involved in the computations of our scheme were {256, 256, 256}, respectively. We verified the network’s performance when the number of participating image blocks decreased by designing the combinations {1, 2, 4}, {2, 2, 4}, and {1, 4, 8}, and we also designed the combinations {1, 6, 36} and {1, 8, 64} to verify the network’s performance when the number of participating image blocks increased. The ablation experiments were performed on the WHU dataset. Likewise, we employed the five-fold cross-validation experiment to validate the authenticity of the networks.

**Figure 12:**
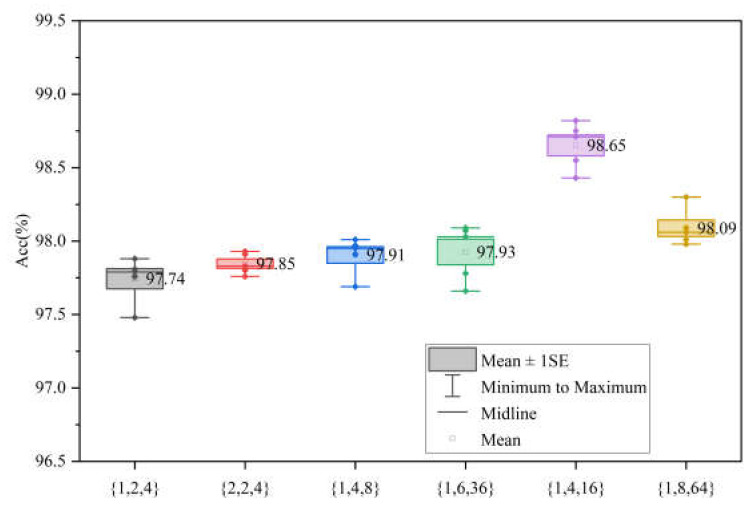
Accuracies of different token combination under five-fold cross-validation experiment on WHU building dataset. The label ‘♦’ represents the accuracy value of each experiment.

As shown in Figure 12, the token combination of {1, 4, 16} achieved a higher average accuracy than those of the other networks, verifying the authenticity of the differences between the different networks.

**Figure 13:**
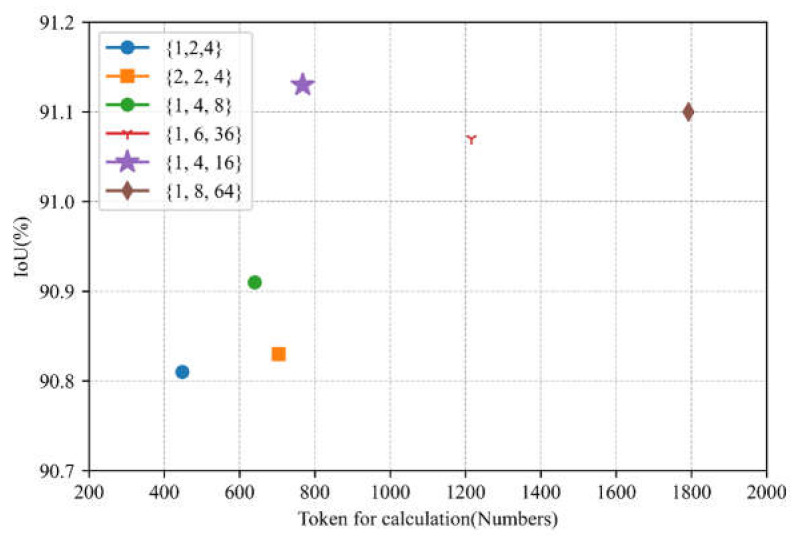
Performance comparison between different image block number combinations.

Subsequently, we utilized the IoU indicator to verify the prediction performance of the test set. The numbers of participating image blocks with the corresponding performance results are shown in Figure 13.

It can be seen in Figure 13 that our network scheme has better performance compared to the other combinations. It is worth noting that the combinations {1, 6, 36} and {1, 8, 64} with more image chunks involved are lower than our network in terms of IoU. It suggests that the network’s performance is not improved by increasing the model participant numbers alone, and also provides further evidence of the superior performance of our combinations on this dataset.

## 6. Conclusions

In this study, an efficient transformer network based on dual-branch fusion of CNN and transformer networks was proposed for efficient and accurate extraction of semantic information of buildings. Traditional convolutional neural networks and transformer networks have some limitations in semantically segmenting buildings in high resolution remote sensing images. The fixed geometric structure and local receptive fields of convolutional neural networks cannot extract global features well; while transformer networks can model global contextual information, they introduce computational redundancies and extract local detail features poorly. To solve these problems, this study reconstructed the transformer structure, and designed the local and global feature extraction transformer module (LGFE). The LGFE was applied to the first three scales of the encoder. The LGFE module consists of the GFE branch and the LFE branch. The GFE branch extracts global features through a DRA module, while the LFE branch obtains fine-grained representations of features. Local and global features are efficiently fused through MLP. In the decoder part, a simple CAM is used for channel dimension enhancement. The network was compared with other mainstream as well as current SOTA methods on the WHU and Massachusetts building datasets, and achieved the best segmentation accuracy. This shows that the network has the ability to extract semantic information of buildings with high efficiency and accuracy, which is of great theoretical and practical significance for the field of HRSI building semantic segmentation. Meanwhile, our proposed transformer module reconfiguration fused GFE branches and LFE branches can be widely applied in more computer vision tasks.

Our network still has limitations. In the ablation experiments, we demonstrated that the values of network hyperparameters work well in the remotely sensed building datasets; however, in the future, we hope that the hyperparameters will be adapted to a specific dataset in order to obtain the best results in a specific task. Meanwhile, we will introduce self-supervised learning and incremental learning in the future to achieve functionality on downstream tasks with unlabeled samples.

## Figures and Tables

**Table 1:** Performance metrics for different networks in the WHU dataset.

Method	IoU	P	R	F1
Segnet	81.88	93.71	74.82	83.71
DeepLabv3+	85.97	94.12	90.23	92.06
HRNet	86.20	94.75	89.99	92.21
TransUnet-S (ResNet-50)	88.49	93.75	87.21	90.36
Swin-T (UperNet)	85.41	93.19	90.34	91.70
PoolFormer-m48 (FPNNet)	87.61	93.75	92.48	93.10
Ours	91.13	95.37	95.06	95.22

**Table 2:** Performance metrics for different networks on the Massachusetts buildings dataset.

Method	IoU	P	R	F1
Segnet	74.37	82.28	69.31	75.24
DeepLabv3+	77.61	88.77	84.30	86.35
HRNet-R48	79.61	88.40	86.40	87.38
TransUnet-S (ResNet-50)	79.76	84.71	78.52	81.50
Swin-T (UperNet)	77.98	87.91	85.49	86.64
PoolFormer-m48 (FPNNet)	77.84	88.96	84.46	86.53
Ours	81.80	89.32	88.32	88.81

**Table 3:** Model parameters and average time per iteration.

Method	Params	Average Time per Iteration	IoU	
			WHU	Massachusetts
Segnet	29.4 M	0.398 s	81.88	74.37
DeepLabv3+	59.0 M	0.749 s	85.97	77.61
HRNet	46.8 M	0.672 s	86.20	79.61
TransUnet	93.3 M	1.206 s	88.49	79.76
Swin-T	60.0 M	0.893 s	85.41	77.98
PoolFormer-M48	73.4 M	0.943 s	87.61	77.84
Ours	59.8 M	0.769 s	91.13	81.80

**Table 4:** Accuracy indicators for different modules. The label ‘√’ indicates that the network has the module.

Method	LFE	GFE	CAM	IoU	P	R	F1
LFE	√			89.92	94.53	94.01	94.26
GFE		√		90.55	95.21	94.55	94.87
LFE + GFE	√	√		91.09	95.31	95.10	95.20
LFE + CAM	√		√	90.42	94.95	94.64	94.80
GFE + CAM		√	√	90.98	95.11	95.14	95.13
GFE + LFE + CAM (Ours)	√	√	√	91.13	95.37	95.06	95.22

## Data Availability

In the aforementioned research, it is mentioned that two distinct datasets pertaining to public building semantic labeling were employed in the study. These datasets include the WHU building dataset and the Massachusetts buildings dataset. Individuals can access these datasets through the following sources: http://gpcv.whu.edu.cn/data/ (accessed on 20 October 2020) for the WHU building dataset, and https://www.kaggle.com/datasets/balraj98/massachusetts-buildings-dataset (accessed on 13 August 2021) for the Massachusetts buildings dataset.
